# Molecular Characterization of *Serratia marcescens* Strain Isolated from Yellow Mealworms, *Tenebrio molitor*, in The Netherlands

**DOI:** 10.3390/insects14090770

**Published:** 2023-09-16

**Authors:** Teresita d. J. Bello Gonzalez, Betty van Gelderen, Frank Harders, Rianka Vloet, Michal Voorbergen-Laarman, Bart de Ruiter, Olga L. M. Haenen

**Affiliations:** 1 Department of Bacteriology, Host Pathogen Interaction and Diagnostic Development, Antimicrobial Resistance Group, Wageningen Bioveterinary Research, Wageningen University Research, P.O. Box 65, 8200 AB Lelystad, The Netherlands; 2 National Reference Laboratory for Fish Diseases, Wageningen Bioveterinary Research, Wageningen University Research, P.O. Box 65, 8200 AB Lelystad, The Netherlands; betty.vangelderen@wur.nl (B.v.G.); rianka.vloet@wur.nl (R.V.); michal.voorbergen-laarman@wur.nl (M.V.-L.); 3 Department of Epidemiology, Bioinformatics and Animal Models, Wageningen Bioveterinary Research, Wageningen University Research, P.O. Box 65, 8200 AB Lelystad, The Netherlands; frank.harders@wur.nl; 4 Independent Researcher, Ringlaan 1, P.O. Box 65, 6961 KJ Eerbeek, The Netherlands; bartderuiter@hotmail.com

**Keywords:** *Serratia marcescens*, mealworms, *Tenebrio molitor*, potential zoonotic, antibiotic-resistancegenes, potential pathogenic bacteria

## Abstract

**Simple Summary:**

*Serratia marcescens* is an important pathogenic bacterium associated with human infections, with remarkably important features related to its intrinsic and acquired antimicrobial resistance. In this study, we aimed to molecularly characterize an *S. marcescens* strain isolated from the skin of rearing-sized yellow mealworms (*Tenebrio molitor*) during an increase in mortality of up to ~30% at a Dutch mealworm farm. We identified the presence of several antimicrobial-resistance genes, all located in the chromosome, and the presence of several virulence genes associated with bacterial invasion. This case demonstrates that *T. molitor* can act as a reservoir and as an alternative path for exposing clinically important antibiotic-resistant bacteria that may affect animals and humans. Although the entomopathogenic activity was not confirmed, it underlines the need to monitor and assess the *One Health* risks of the bacteria present in individual insect farms, before insects and their products may enter the feed and food chain.

**Abstract:**

Insect culture has developed rapidly worldwide; it faces important security and safety control issues, including animal infections and disease development. In the Netherlands, in 2021, a ~30% mortality of mealworms, *Tenebrio molitor*, occurred at one farm, where over-humid sites in the substrate were observed. Bacterial cultures from both the external and internal partsof fry and larger mealworms were identified by MALDI-TOF to predominantly *Serratia marcescens, Staphylococcus xylosus* and *Staphylococus saprofyticus*. Due to the important role of *S. marcescens* as a potential zoonotic bacterium, we performed a molecular characterization of the isolated strain. Genomic analysis showed a multidrug-resistant *S. marcescens* isolate carrying a *tet* (41), *aac* (6′)-*Ic*, and *bla*SST-1 chromosomal class C beta-lactamase-resistantgenes, all located on the chromosome. Additionally, several virulence genes were identified. The phylogenetic tree revealed that the *S. marcescens* strain from this study was similar to other *S. marcescens* strains from different ecological niches. Although the entomopathogenic activity was not confirmed, this case demonstrates that *T. molitor* can act as a reservoir and as an alternative path for exposing clinically important antibiotic-resistant bacteria that can affect animals and humans. It underlines the need to keep management factors optimal, before insects and their products enter the feed and food chain.

## 1. Introduction

Insect culture is an important and fast-growing sector, as it provides a new source of animal protein for feed and food. One of the cultured insect species is the larvae of *Tenebrio molitor* (Coleoptera: Tenebrionidae), a species of darkling beetle, the so-called mealworm, cultured with success for the feed of cultured fish, poultry, and for food [1]. During its lifespan, this mealworm has gone through several stages of development, including the egg, larvae, pupa, and adult phases. Between their different life stages, the morphology of the mealworm varies drastically [2].

Several abiotic factors, such as temperature, humidity, and oxygen concentration during their rearing process, as well as biotic factors, such as the population density present per stage, parental age, and feed quality, play an important role in their growth and development [3]. Therefore, careful consideration and good management practice must be applied during their entire rearing process.

In the Netherlands, the insect culture branch has been developing exponentially over the last decade, including the culture of larvae of the yellow mealworm *T. molitor*, for feed and food [4]. For a *T. molitor* culture to grow, several environmental conditions, including light, temperature, relative humidity, and oxygen, need to be controlled, monitored and maintained to ensure the development of the mealworm. Likewise, the monitoring of the nutrient content present in the feed and the ratio of wet feed needed as a water source should be constantly controlled to secure an optimal mealworm production in orderto be able to apply an intervention when these conditions change [4]. Regarding the environmental conditions, *T. molitor* mealworms are best cultured in a dark environment which haslimited access to direct incoming light and in a temperature range of between 27 and 31 °C, since they produce heat themselves due to their metabolic activity [4,5]. This particular characteristic may cause an increase in the temperature inside the crate, containing substrate and mealworms, of up to ~8 °C and it will depend, on the insect density in the crates and the mealworm development stage [4]. Their ability to tolerate temperature depends on their development stage, duration of exposure, and the influence of humidity in their environment. The recommended levels of relative humidity range between 60 and 70%. Several studies have shown that mealworms are able to survive with relative humidity up to 98% [6], while eggs are susceptible to low relative humidity (12%) [4]. Higher relative humidity in the production of *T. molitor* mealworms is considered not ideal since this could cause mite proliferation and growth of molds in the substrate, whereby the condensation of water at colder walls could occur, and may be the cause of over-humid sites in the substrate [4]. At this point, a third critical factor to control and monitor is the air refreshment and air movement system needed to get rid of the CO_2_ produced by the larvae, allowing for the fresh oxygen air to flow. The refreshment of the air is beneficial for the removal of fine dust particles and fungi spores as well as for the health of the farmers due to potential allergic effects. When the above-mentioned environmental conditions are suboptimal, unwanted organisms can replicate in their environment [4].

Two types of microbial communities could influence the farming environment: the community intrinsically associated with the insect and the one introduced during the rearing process [7]. An important variation in these microbial communities is dependent on the feed substrate and frass. Osimani et al. (2018) showed that feed and frass could serve as a potential route for microbial contamination, including several members of *Enterobacterales* and Acid Lactic Bacteria [8]. Moreover, Jensen et al. (2020) showed that the presence of *Salmonella enterica* serovar *Typhimurium* can be detected in the larvae after 14 days of exposure to a contaminated substrate, highlighting the importance of avoiding the introduction of *Salmonella* via a contaminated substrate during the production [9]. Given the fact that the environmental conditions during the rearing process, such as temperature, are close to the human temperature of 37 °C, potential zoonotic bacteria may develop as well, especially if the management to prevent food and veterinary safety issues is suboptimal.

The increasing number of farms in the Netherlands is associated with the need to balance the aforementioned management factors, including the control and monitoring of the farm environmental conditions, as well as the use of uncontaminated substrates. Therefore, in 2018, the Dutch Council on Animal Affairs stated the need for monitoring and research on the insect health and safety risks for animals and humans regarding this new protein entering the feed and food chain [10]. This is especially relevant since insect culture uses non-sterile leftover streams from the food industry, although with strict rules to prevent veterinary, food and *One Health* safety issues [11]. Nevertheless, several diseases and pathogens have been identified in farmed insects [12].

One of the pathogenic bacteria that has been identified in insects is *Serratia marcescens*. *S. marcescens* is a Gram-negative motile Proteobacteria, a member of the order Enterobacterales, commonly associated with opportunistic infections in humans, and in various animal species [13,14]. In humans, the bacterium has been described to cause diarrhea in children in low-income countries [15], endocarditis, sepsis, infections of prosthetic heart valves [16], and nosocomial infections, whereby both hand-to-nose contact (contact zoonosis) and ingestion of the bacterium (food zoonosis) may infect humans [17,18,19]. In poultry [20,21], *S. marcescens* is considered to be a commensal respiratory bacterium. In aquaculture, *S. marcescens* has been isolated as an opportunistic pathogen of tilapia, *Oreochromis niloticus* [22], with a single case of ampullary system infection and septicemia in a bonnethead shark, *Sphyrna tiburo* (L.) [23], and in elkhorn coral, *Acropora palmata*, causing white pox disease [24].

*S. marcescens* is ubiquitous in the environment. It has been isolated from water, soil, plants and insects [25]. In insects, it has been identified in members of Coleoptera in various geographic regions in the world [26,27,28,29,30], including in the adult boll weevil *Athonomis grandis* [31]. Other host examples of *S. marcescens* are the verde plant bug *Creontiades signatus* (Distant) in S-Texas [32], *Protaetia brevitarsis seulensis* (Kolbe) in Korea [33], together withvarious other insects, like silkworms [34] and lepidoptera [35,36,37]. The strain Ss1 of *S. marcescens* has been described in mites, *Metaseiulus occidentalis* (Acari: Phytoseiidae) [38], and as a new pathogen for honey bees, *Apis mellifera* [39]. Sometimes, high mortality may occur, for instance, in caterpillars of the beet armyworm, *Spodoptera exigua* (Spodoptera), and approximately 50% in the experimentally orally infected adult boll weevil, *Athonomis grandis* [31,40].

*S. marcescens* has also been described as a persistent pathogen of *T. molitor* by Dupriez et al. (2022), only with a low increase inmortality (2%) in their study [41]. Clinical signs of disease by *S. marcescens* in a pupa of *T. molitor* show it to become pinkish in color [42]. Pineda et al. (2015) described that the larvae of the scarab, *Phyllophaga blanchardi* (Coleoptera), demonstrated that the *S. marcescens*-infected insects were lethargic and had a brown color compared with the beige-coloured *S. marcescens*-negative insects; indicating a darkening in color of the infected insects [29].

*S. marcescens* is also known as a beneficial bacterium as it has been used for the digestion of chitin when added to chicken feed since it produces chitinase [43,44], which chickens do not have. Moreover, some antibacterial properties have been identified in dairy, and seen to act against *Yersinia enterocolitica* and *Listeria monocytogenes* [45].

In this study, we describe the molecular characterization of a *Serratia marcescens* strain isolated from yellow mealworms, *T. molitor*, from a Dutch insect farm, showing lethargic and black dead larvae and a mealworm mortality of 20–30%. The entomopathogenic activity of the *S.marcescens* strain was not evaluated in this study.

## 2. Materials and Methods

A Dutch farmer of yellow mealworms, *T. molitor*, reported to the local veterinarian an increased mortality in the second phase of the rearing, showing lethargic, black and even dead larvae. The mealworm mortality was ~20%.

The veterinarian collected four samples from fry and older mealworms, comprising the external and internal parts of a mealworm, respectively, using sterile disposable loops. The loop samples were individually plated directly (non-diluted) onto Plate Count Agar (PCA) (Oxoid) and Sheep Blood Agar (Tryptone Soy Agar with 5% Sheep Blood) (Oxoid) plates, respectively. The agar plates were kept at room temperature (appr. 20 °C) for two days, after which the plates were sent to the Wageningen Bioveterinary Research (WBVR) laboratory for bacterial identification.

A second consultation by the veterinarian to this insect farm, 24 days after the first visit, revealed that the mortality in the complete rearing phase population of the yellow mealworms had reached ~30%. No samples were taken at that date.

From the culture plates received at WBVR, pure cultures were made on fresh Tryptone Soya Agar plates supplemented with 5% Sheep Blood and incubated for two days at 20 °C in an incubator. Purified colonies were retrieved, and bacterial identification was performed on single colonies using MALDI-TOF (matrix assisted laser desorption/ionization time-of-flight, Bruker Nederland BV, Leiderdorp, The Netherlands), according to the procedure described in the paper of Jansson et al. (2020) [46].

Whole-genome sequencing analysis was performed using a fresh and pure culture of an *S. marcescens* strain isolated from the outside of the mealworm only. DNA was isolated and purified using the Qiagen DNA isolation BloodKit, and the DNA concentration was measured with a CLARIOstar Plus (BMG Labtech). The isolated DNA was used for library preparation using Illumina DNA Prep. The constructed NGS library was PE150-sequenced on a NovaSeq 6000. After demultiplexing, raw read data from paired-end sequencing were quality checked with the FastQC tool (v.0.11.6). BBtools [47] was used for adapter trimming, polished reads were used for constructing a draft genome using SPAdes (v.3.12.0) software, and QUAST software (v.5.2.0) was used for the quality of assembly. The assembled genome was analyzed using tools from the “Center for Genomic Epidemiology” (CGE) website, Prokka software (v.1.13) for annotation, and Abricate software (v.0.8 8) for screening for antimicrobial resistance and virulence gene. The sequencing data of the *S. marcescens* strain were deposited at NCBI/GenBank under the accession numbers SAMN37283987. The genes associated with virulence factors of *S. marcescens* were identified and annotated using DFAST NCBI BLAST (https://blast.ncbi.nlm.nih.gov/Blast.cgi accesed on 23 May 2023).

A phylogenetic analysis was performed to identify related species of the *S. marcescens* isolate. A total of 14 *S. marcescens* strains were retrieved from the GenBank database (http://www.ncbi.nlm.nih.gov/blast accesed on 30 May 2023) to compare with our isolate. The tree was constructed using the MEGA 7.0 [48] software and the Neighbor-Joining method [49]. The evolutionary distances were computed using the p-distance method [50] displayed in the units of the number of base differences per site. The multiple alignments of the sequences were performed by ClustalW Omega (http://www.ebi.ac.uk/Tools/msa/clustalo accesed on 30 May 2023).

## 3. Results

### 3.1. Characteristics of the T. molitor Specimen

The anamnesis, as reported by the veterinarian (pers. comm.), mentioned that locally on the farm, the rearing conditions were too wet for the mealworms *T. molitor*; in particular, over-humidity in the substrate was observed. At these sites in the system, rotting was recorded. The total mortality of *T. molitor* larvae was estimated to be ~30%.

#### 3.1.1. Bacterial Identification

*S. marcescens* was isolated and identified from the larger mealworms outside (one isolate) and inside (two isolates) only. Unfortunately, only the *S. marcescens* isolate obtained from the outside of the mealworm was retrieved and further characterized in this study.

Furthermore, *Staphylococcus xylosus* was identified from the mealworm fry (one isolate) and larger mealworms inside (one isolate) and *S. xylosus* (one isolate) and *Staphylococcus saprofyticus* (one isolate) from the outside of the larger mealworms only. These other bacterial species were not further analyzed.

#### 3.1.2. Molecular Characterization of *S. marcescens*

**Figure 1:**
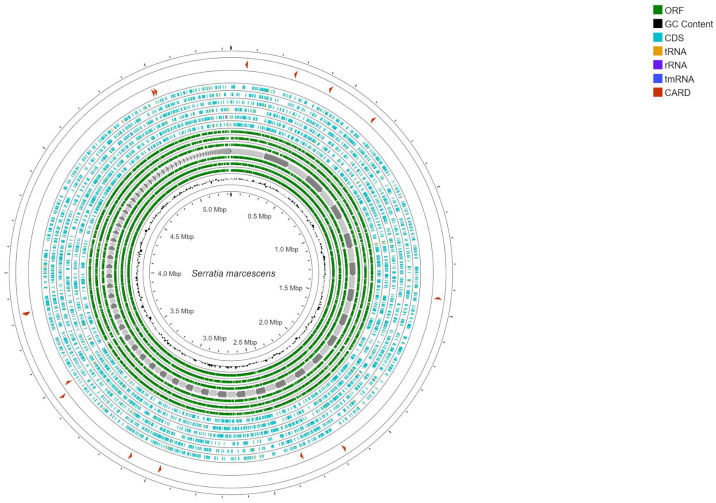
Map of the *S. marcescens* strain isolate from *Tenebrio molitor*. From the outermost circle towards the center, the positions of the antibiotic-resistance genes are indicated (in red), CDSs (blue) with inclusion of the tRNA, rRNA and tmRNA (orange, purpure and blue, respectively), ORF (green), and GC content (black). CGview software was used to construct the genome map.

The complete genome of *S. marcescens* comprised approximately 5,333,490 bp, carrying 4,881 of the coding DNA sequence (CDS), with an overall G+C content of 59.21%. The N50 of the *S. marcescens*-assembled genome was 65,649 bp. A total of 3 rRNA and 85 tRNA genes were identified. The *tet* (41) tetracycline resistance, *aac* (6′)-*Ic* aminoglycoside resistance, and *bla*SST-1 (STR-2) chromosomal class C beta-lactamase-resistance genes were identified. In addition, we found several genes encoding for important efflux pumps that were associated with metal resistance (*smd*A, *Smd*B, *Smf*Y and *Ssm*E, *sde*B and *has*F), a global regulator that represses MdtEF in macrolides (CRP), two antibiotic efflux pump *mdt*G members of the major facilitator superfamily of transporters (MFS) associated with fosfomycin resistance, and the *acr*B member of the resistance-nodulation-division (RND) family of transporters associated with fluoroquinolone resistance. A point mutation on protein gene *rpo*B resulting in resistance to rifampicin was identified (Figure 1). The bacterial virulence factors *rsm*A (ribosomal RNA small subunit methyltransferase) *flh*A, *flh*B, *flh*C, and *flh*D (flagellar biosynthesis protein and transcriptional regulator); *shl*B_1 and *shl*B_2 (hemolysin activator proteins); and *bsm*A (biofilm-peroxide-resistance protein), *Rss*B (two-component system response regulator), *fim*B (type 1 fimbriae regulatory protein), and *omp*X (outer membrane protein), involved in the bacterial invasion, were identified.

#### 3.1.3. Phylogenetic Tree

**Figure 2:**
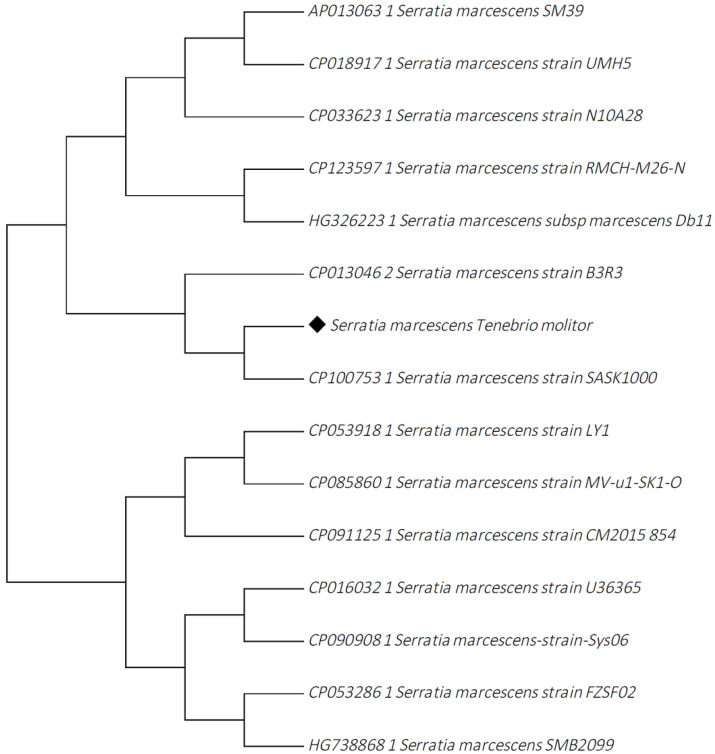
Phylogenetic relationships of the *S. marcescens* strain of this study, isolated from mealworms, *T. molitor* (black), with other related *S. marcescens* strains sequences, obtained from the GenBank database. The analysis involved 15 nucleotide sequences. All positions containing gaps and missing data were eliminated. There was a total of 5,113,802 positions in the final dataset. Accession numbers of the sequences are shown in the tree. The optimal tree with the sum of branch length = 5.55112989 is shown.

The phylogenetic tree shows that our isolated strain had high similarity to other *S. marcescens* strains, particularly with isolates obtained from *Drosophila*, fruit flies (Db11), *Apis mellifera*, Western honey bee (N10A28), *Blattella germanica*, German cockroach *(*RMCH-M26-N), and human clinical isolates (UMH5, SM39) (Figure 2).

## 4. Discussion

Since 2010, the agricultural branch of insect farming for food and feed has increased in the Netherlands and Belgium, showing a very steep growth internationally [1]. Although a few researchers have focused on the veterinary and food safety risks of insect farming for protein production in the last decade, still much is unknown regarding diseases and possible zoonotic risks [10].

In this study, we observed an increase in mortality in a population of yellow mealworms, *T. molitor*, at a single Dutch insect farm. Three different bacterial species were identified from the two stages of mealworms (fry and/or bigger worms).

From these, we molecularly characterized the *S. marcescens* strain isolated only from the outside of the bigger mealworms. We were unable to perform a molecular characterization of the strain isolated from the inside of the mealworm because this isolate was not further recovered. With only one isolate available for our study, we recognized that drawing meaningful conclusions regarding the entomopathogenic activity of the *S. marcescens* strain isolated from the outside of the bigger mealworms would be challenging, considering that the original source of the strains is still unknown. This limitation underscores the importance for comparing both isolates (inside and outside) to determine their similarities and the ability to explore the original cause of the increased mortality observed. Future research in this area should prioritize obtaining larger sample sizes to overcome this limitation and provide more comprehensive insights into the subject matter such as the inclusion of an experimentally induced infection with the isolates in *T. molitor*. Despite this limitation, we consider the *S. marcescens* isolated from *T. molitor* as a potential pathogenic bacterium associated with human infections, i.e., a bacterial species with zoonotic potential [14,16]. Moreover, *S. marcescens* is known as an opportunistic bacterial pathogen of insects, poultry, fish, and calves. [13,18,20,21].

This study was carried out to estimate the possible impact of the *S. marcescens* isolate on insect farming for food and feed. The Council on Animal Affairs (Raad voor Dierenaangelegenheden) of the Netherlands [10] recommended in their report to monitor insect farms regularly for food and veterinary safety during the different stages of insect development in various systems per farm, and over a period of time, to be able to assess the risk for the presence and effect of potential food and veterinary pathogens, related to *One Health*.

For insects, stress factors are a basis for acquiring infections, such as sudden changes in their environment, such as, a decline in the temperature, which can affect the weight gain and food conversion efficiency, when insects are injured, or when there is cannibalism in the insect group [26,30,51]. In this particular case, at least suboptimal climate conditions were relevant, namely, over-humid sites in the substrate, as reported by the owner to the local veterinarian. Rotting places in the substrate were seen by the farmer. We hypothesize that these conditions may have contributed to the increased mortality observed. Urs and Hopkings demonstrated that moisture conditions could affect the growth rate and development of *T. molitor* larvae [52]. Moreover, in the study of Dupriez et al. (2022), the strain of *S. marcescens* used in their experimental setup identified in the feed and feces of *T. molitor* was causing only a ~2% increase in mortality. Therefore, the authors considered that the presence of *S. marcescens* in mealworm production could be indicative of a lower sanitary status [41]. The presence of suboptimal environmental conditions was the main issue in this case. It can be prevented through good husbandry management, like the monitoring, controlling and maintenance of the environmental conditions; the use of controlled and monitored substrate; and wet/dry feed and frass. In this case, the over-humid sites caused rotting places in the substrate, in which a multi-bacterial infection may have quickly developed, not only of *S. marcescens*, but also of other opportunistic bacteria.

For insect farmers, finding and maintaining a balance between the environmental conditions such as temperature, humidity and air quality in the insect culture unit, and the substrate, is a challenge. A preventive method to test for the presence of bacteria like *S. marcescens* in the meconium of lepidopterous heliothines has been described [53]. When this test is positive, it provides an idea of possible disease problems under certain circumstances. When this test is negative in statistically significant monitoring, it can be used as a basis for specific pathogen-free (SPF) insect rearing. Treating with chemicals or antibiotics against such bacteria at an insect farm is not an option because these will accumulate into residues, which would enter the food and feed chain with the harvested insects and frass. It is important to consider that the choice of the substrate used during the rearing process influences the risk of introducing chemical and microbiological contaminants, thereby reducing the veterinary and food safety of the insect products. It is also important to realize that there may be more bacteria of risk present at the insect farm and in the harvestable insect than that covered in the food safety legislation and standard HACCP testing [54], as proven in our study in a single mealworm farm. Therefore, critical control of the nature of the substrate used for insect rearing is essential to assess safe insect production and bioconversion use.

Since, in Western societies, there is a wish to produce insects more circularly and sustainably, using new leftover streams as a substrate, like out-of-date supermarket waste, kitchen waste, chicken manure, and dried category III slaughter waste, is currently being explored. Although these leftover streams are not yet accepted, as a substrate to grow insects on, this has become even more relevant, and those safety risk assessments are in progress, such as the project SAFE INSECTS, led by Wageningen Food Safety Research of Wageningen University and Research, the Netherlands [55].

The genomic analysis of the *S. marcescens* strain indicated the presence of several antibiotic- resistance genes. Similar resistance genes were identified from clinical and environmental *S. marcescens* isolates during an outbreak in a neonatal intensive care unit in Italy [56]. The chromosomal aminoglycoside-resistance gene *aac* (6′)-*Ic* confers resistance to gentamycin, tobramycin, netilmicin, amikacin, and the *tet* (41) tetracycline protein efflux pump, which are all found in clinical and environmental *S. marcescens* strains [57,58]. All of the resistance genes identified in the genome of *S. marcescens* were present in the chromosome and not acquired by gene horizontal transfer. Similar resistance genes were found in the chromosome of an *S. marcescens* isolate obtained from *Drosophila melanogaster* [59].

The virulence genes identified in the genome of *S.marcescens* might correlate with the ability of the bacteria to penetrate and colonize the mealworm’s gut, followed by penetration of the gut cell wall to access the body cavity. In particular, the flagellar gene, biofilm formation, and hemolysin have been found to play an essential role in the pathogenicity in bacteria [60,61]. Similar virulence factors have been identified in isolates from diseases in fish (Tilapia, *O. niloticus*), *D. melanogaster*, and clinical (blood culture) *S. marcescens* strains [59,62]. The phylogenetic analysis of this study shows that our isolate is related to several other *S. marcescens* isolates from other insect species, but is also similar to human clinical isolates. Considering the broad range niche of *S. marcescens*, an important potential pathogen, our results suggest that *T. molitor* has the ability to capture, maintain, and possibly spread these bacteria across the entire rearing population. However, we were unable to perform further analysis that could elucidate the origin of this isolate, the comparison between *S. marcescens* isolates from inside and outside of the mealworm, and to determine the entomopathogenic activity of this strain. Apart from the high mortality in the *T. molitor* population, the recovery of this pathogenic strain in this single case highlights the need to better understand, where, when, and how opportunistic pathogens such as *S. marcescens* can infect, invade the host, and disseminate within and across animal species and/or humans via the feed and food chain.

The bacteriological risk of cultured insects to humans and other vertebrates relates strongly to the insect microbiota, which is dependent on the rearing conditions, wet/dry feed, handling, and the processing of insects during the production cycle, although *S. marcescens* is also ubiquitous in the environment, soil, and plants. Therefore, the presence of a zoonotic pathogen such as *S. marcescens* in the substrates used to grow insects could potentially lead to insects acting as a vector for these bacteria. In this context, the environmental conditions and substrate used to feed the insects have a direct influence on the insect microbiota (internally and externally) and are influenced by the hygienic conditions present at the farm environment [54]. The test mentioned above [53] may help to monitor and thereby prevent the growth and development of this bacterium at an insect farm.

The risk of the presence of the three isolated bacterial species identified in this study in the food chain and the consumers is considered low, according to Vandeweyer et al. (2017), Lenaerts et al. (2018), and Kröncke et al. (2018, 2019), due to the pre-treatment of the mealworms before entering the food chain [63,64,65,66]. The most common method is the drying process, which can be performed in various ways. Vandeweyer et al. (2017) tested the blanching and industrial microwave methods and concluded that blanching for 10, 20 or 40 s can result in a sufficient microbial reduction, i.e., at temperatures up to 72 °C after 20 min of drying; this killed most non-spore-producing bacteria, but did not kill the bacterial spores [63]. Lenaerts et al. (2018) studied the suitability of microwave drying as an alternative to the freeze-drying method for *T. molitor* larvae, and confirmed that the microwave-drying method could be a good alternative for the maintenance of the nutritional properties of the larvae, although they did not test for bacterial survival [64]. Kröncke et al. (2019) further analyzed the oven-drying method on *T. molitor* larvae at 60 °C under vacuum conditions for 24 h, and drying via a rotating rack oven at 120 °C for 1 h [66]. Moreover, Kaira et al. (2015) demonstrated that a wide range in temperatures and pH levels allows for a psychrotolerant strain of *S. marcescens* to survive between 4 and 45 °C [67]. In addition to these pre-treatment options to dry harvested mealworms in practice, we also know from Vandeweyer et al. (2017) [63] that Enterobacteriaceae are eliminated; since *S. marcescens* is a member of the Enterobacteriaceae, the risk of insect consumers acquiring an *S. marcescens* infection when eating dried mealworms by the above-mentioned methods will be low. Both *Staphylococcus xylosus* and *S. saprofyticus* can grow between 6.5 and 46 °C; since they are no spore-forming bacteria, they can be eliminated at temperatures above 65 °C [68].

Based on the above, the risk for acquiring a zoonosis by *S. marcescens* is more present at insect farms and insect processing units through direct skin contact with a substrate, insects, and through possible inhalation than in human food; thus, consideration should be given to professionals working at these sites. Good prevention should therefore always be in place, like good hygiene protocol and practice [69].

The risk of zoonosis in the farming of cold-blooded animals has been reported in aquaculture and ornamental fish branches [70], especially in bacteria that tolerate temperatures close to the human temperature. Like in other animal production branches, it is important to protect the insect farmer and processor. Since *S. marcescens* may infect humans via hands to the nose and cause nosocomial infections [19], and since some of the smaller insect farmers have direct hand contact with the substrates and insects at their farm, it is crucial to protect the insect farmer processor against contact zoonosis through hygienic precautions, like protective clothing and gloves, as described in the hygiene standards of the International Platform of Insects for Food and Feed [69].

Although a single isolate from one disease case in one yellow mealworm farm was molecularly characterized, our findings underline the need to monitor and assess the *One Health* risks of potential pathogenic bacteria at every individual insect farm in the various insect developmental stages, the substrate, in time, and per unit before the harvested live insects safely enter the feed and food chain. 

## 5. Conclusions

We identified *S. marcescens* from a Dutch mealworm farm of *T. molitor* as the possible main cause of a 30% increase in mealworm mortality. Although we only molecularly studied the *S. marcescens* isolated from the outside of the mealworm, and did not evaluate and prove the entomopathogenic activity of these isolates, our study indicates that suboptimal environmental conditions and management factors, like over-humid sites in the substrate, may have facilitated this multi-bacterial infection by *S. marcescens* and other opportunistic bacteria. The presence of several antibiotic-resistance genes and virulence factors highlight the importance of considering *T. molitor* as a reservoir and as an alternative path for the exposure of clinically important antibiotic-resistant bacteria, such as *S. marcescens*, which could potentially affect animals and humans when good management practices are not in place. An infection with *S. marcescens* can be considered a potential risk for insect farmers and processors who have direct contact with the infected insects. However, the risk for the food chain will be small, since the insects and products will be pre-treated to eliminate these bacteria before they enter the food and feed chain for consumption. 

Our study underlines the need to monitor and assess the food safety, veterinary safety, including the *One Health* risks of potential pathogenic bacteria present in individual insect farms, through the standard monitoring of all individual insect farms’ environmental conditions, substrates, and frass, before insects and their products may enter the feed and food chain, and through strict hygiene standards.

## Data Availability

The sequencing data of the *S. marcescens* strain have beenwere deposited at NCBI/GenBank under the accession numbers SAMN37283987.
